# Ammonia-oxidizing archaea are integral to nitrogen cycling in a highly fertile agricultural soil

**DOI:** 10.1038/s43705-021-00020-4

**Published:** 2021-06-01

**Authors:** Laibin Huang, Seemanti Chakrabarti, Jennifer Cooper, Ana Perez, Sophia M. John, Samira H. Daroub, Willm Martens-Habbena

**Affiliations:** 1 Fort Lauderdale Research and Education Center, Microbiology and Cell Science, University of Florida, Davie, FL USA; 2 Everglades Research and Education Center, Soil and Water Sciences, University of Florida, Belle Glade, FL USA

**Keywords:** Microbial ecology, Biogeochemistry

## Abstract

Nitrification is a central process in the global nitrogen cycle, carried out by a complex network of ammonia-oxidizing archaea (AOA), ammonia-oxidizing bacteria (AOB), complete ammonia-oxidizing (comammox) bacteria, and nitrite-oxidizing bacteria (NOB). Nitrification is responsible for significant nitrogen leaching and N_2_O emissions and thought to impede plant nitrogen use efficiency in agricultural systems. However, the actual contribution of each nitrifier group to net rates and N_2_O emissions remain poorly understood. We hypothesized that highly fertile agricultural soils with high organic matter mineralization rates could allow a detailed characterization of N cycling in these soils. Using a combination of molecular and activity measurements, we show that in a mixed AOA, AOB, and comammox community, AOA outnumbered low diversity assemblages of AOB and comammox 50- to 430-fold, and strongly dominated net nitrification activities with low N_2_O yields between 0.18 and 0.41 ng N_2_O–N per µg NO_x_–N in cropped, fallow, as well as native soil. Nitrification rates were not significantly different in plant-covered and fallow plots. Mass balance calculations indicated that plants relied heavily on nitrate, and not ammonium as primary nitrogen source in these soils. Together, these results imply AOA as integral part of the nitrogen cycle in a highly fertile agricultural soil.

## Introduction

Human activity has significantly altered the global nitrogen cycle within the past century. Nitrogen inputs from industrial sources (e.g. nitrogen fertilizer and atmospheric deposition) and crop N fixation together exceed inputs from natural nitrogen fixation and accelerate the nitrogen cycle globally.^1,2^ Nitrification, the microbial oxidation of ammonia to nitrite (NO_2_^−^) and nitrate (NO_3_^−^) is a central step in the nitrogen cycle and provides the substrates for the nitrogen removal processes denitrification and anaerobic ammonia oxidation to N_2._^3,4^ However, nitrification rates are particularly high in agricultural systems, deemed to limit crop nitrogen use efficiency, leading to nitrogen leaching and eutrophication in coastal zones, and increasing the global inventory of nitrous oxide (N_2_O).^5–8^

Ammonia oxidation, the rate-limiting step of nitrification, is now known to be driven by a trifecta of phylogenetically and physiologically distinct microorganisms including ammonia-oxidizing bacteria (AOB), ammonia-oxidizing archaea (AOA), and complete ammonia-oxidizing (comammox) bacteria within the bacterial genus *Nitrospira.*^9–12^ AOA often numerically dominate over AOB in pristine neutral and acidic soils.^13–15^ In contrast, in most fertilized agricultural soils AOB are deemed the predominant ammonia oxidizers and hence primarily responsible for N-loss and N_2_O emissions.^15–19^ Comammox ammonia oxidizers are widespread in terrestrial systems such as soils, wetlands, freshwater sediments, groundwater aquifers, and water distribution systems.^20–22^ However, their significance in agricultural systems remains poorly understood. To date, the only isolated comammox strain has been shown to possess a low *K*_m_ for ammonia and low N_2_O yield similar to AOA.^23,24^ However, mechanistic insights into the physiological and environmental controls on activity and N_2_O emissions of either group in mixed communities remain scarce.^18,19,25–27^

Recent studies have used 1-octyne as a selective inhibitor targeting AOB in nitrification potential^28–30^ and net nitrification assays,^31,32^ and to discern activities and N_2_O yields of AOA and AOB in soils.^31,32^ These studies suggested a prevalence of AOA activity at low ammonia input and stimulation of AOB by ammonia fertilization.^28–32^ Emissions of N_2_O were lower for AOA than AOB.^31,32^ Although the use of ‘selective’ inhibitors has many potential pitfalls, these pioneering studies provided mechanistic evidence for niche separation of AOA and AOB, and associated lower N_2_O yields from AOA, and potential for N_2_O emission reductions from agricultural soils by favoring AOA activities through management.^18,31^

In soils, the activity of AOA has been linked to organic matter mineralization with low fluxes of NH_4_^+^.^33–35^ We hypothesized that organic matter mineralization-linked activity of AOA and associated low N_2_O yield should manifest in agricultural soils with high organic matter mineralization rates and high N fluxes. We therefore aimed to determine nitrification rates and N_2_O yields in the absence of fertilizers or selective inhibitors, thereby potentially providing independent evidence for such AOA niches and low N_2_O yields. Furthermore, we hypothesized that competition between plants and nitrifiers for reduced nitrogen would curb nitrifier abundances in cropped soils, and conversely, fallow treatments would naturally enrich nitrifiers. To test these hypotheses, we selected one of the most fertile agricultural soils within the continental United States with organic matter contents between 60 and 85% and carbon and nitrogen mineralization rates high enough to sustain sugar cane production without application of nitrogen fertilizer.^36,37^ These soils located in the Everglades Agricultural Area (EAA) in southern Florida were claimed from freshwater marshes of the Florida Everglades in the early 1900s for agricultural use. Previous research demonstrated that these soils harbor between 1.7 and 3.6% *Nitrososphaera*-related *Thaumarchaeota* based on relative frequency of 16S rRNA genes.^37,38^ However, no detailed studies on nitrification have been conducted. Our results show that organic matter decomposition-linked AOA activity with low N_2_O yields vastly dominates over AOB and comammox activities in these soils, and suggest interactions between microbial nitrogen mineralization, AOA activity and plant nitrogen uptake, as well as nitrogen leaching within the greater Everglades ecosystem.

## Materials and methods

### Net nitrification and nitrification potential rates

Soil samples were collected in December 2017 in the EAA near Belle Glade, FL (Supplementary Fig. 1). One unmanaged native plot (plot 1) and four agriculturally managed plots (plot 2–5) in close proximity to each other were sampled. Plots 2–5 had been cultivated with sugar cane for 2 (plot 5) or 3 (plot 2–4) years. Subsequently, plot 2 was planted with spinach from January to May 2017, followed by a 28-week fallow period before sampling. Plot 3 was planted with sweet corn from January to May 2017, followed by flooding and rice cultivation from May until October 2017, and an 8-week fallow period from October to December 2017. Plot 4 was covered with sweet corn from January to May 2017, also followed by a 28-week fallow period until sampling in December 2017. Plot 5 was cultivated with sugar cane for a third consecutive year until harvest in November 2017, 1 week before sampling. The unmanaged native plot 1 was covered with a mixed community of annual and perennial plants, including elderberry (*Sambucus canadiensis*, ~60% cover), ragweed (*Ambrosia artemisiifolia*, ~30%), knotroot foxtail (*Setaria parviflora*, ~5%), Columbus grass (*Sorghum almum*, ~5%), common lantana (*Lantana camara*, ~1%), common pokeweed (*Phytolacca americana*, ~1%).

Three composite bulk soil samples were collected between 50 and 200 m apart in each plot. Each composite sample consisted of nine individual 10-cm topsoil cores collected within a 10 × 10 m^2^ area, combined, and transported to the laboratory on ice. Net nitrification and N_2_O production rates were determined in 10 g soil microcosms according to Hink et al.,^32^ at 28 °C with incubation times of 144 h; rates were calculated from linear regression of NO_2_^−^ + NO_3_^−^ accumulation and N_2_O headspace concentrations over time (see Supplementary Materials and Methods for detailed description of soil selection, sampling, physicochemical soil analyses, and rate measurements).

Nitrification potentials were assessed using a modification of the method by Hart et al.^39^ Preliminary experiments conducted on samples collected 2 months earlier showed lower nitrification potentials than net nitrification using the standard phosphate buffer method. We therefore replaced phosphate buffer with synthetic freshwater Crenarchaeota medium. This medium has been used routinely to grow AOA and AOB (e.g.,^40^) and also supported growth of comammox enrichments (S.C. and W.M.H., unpublished observations). Field-moist soil (1.0 g) was added to 20 ml media in 50 ml Falcon tubes, vortexed briefly, and incubated at 28 °C. Potential nitrification rates were determined from linear regression of NO_2_^−^ + NO_3_^−^ accumulation over the first 48 h incubation. (See Supplementary Materials and Methods for further details).

### Molecular analyses

Soil samples for molecular analyses were placed on dry ice immediately after sampling in the field and stored at −80 °C until further processing. Samples (~5 g) were homogenized by grinding under liquid nitrogen using mortar and pestle. DNA was extracted from 0.25 g (wet weight) soil using the DNeasy PowerSoil kit (Qiagen, Germantown, MD) following manufacturer’s recommendations. Samples were treated by bead beating twice for 30 s at 4.0 m/s in a FastPrep-24 bead beater (MP Biomedical, Irvine, CA). DNA quality and quantity was assessed by UV/Vis spectroscopy using a NanoDrop ND-1000 (Thermo Scientific, Waltham, MA). DNA concentrations were between 40 and 168 ng/μl with 260/280 nm ratios >1.7 from all samples. DNA samples were stored at −80 °C until use.

### Clone library analysis of archaeal, bacterial, and comammox *amoA*

Amplicon clone libraries were generated for each plot and ammonia oxidizer group. For comparison of comammox diversity an additional comammox *amoA* gene clone library was prepared from a surface soil sample collected from intact Everglades wetlands site SRS3 in December 2019. Gene fragments of target *amoA* were PCR-amplified using previously described primers for archaeal,^41^ bacterial^42^ and comammox clade A and clade B,^20^ purified, cloned, amplified, and Sanger-sequenced. PCR reactions for comammox clade B were negative from all five plots and site SRS3, therefore no sequencing of PCR products was conducted. All obtained sequences were trimmed using sangerseqR^43^ and manually aligned and quality-checked using the ARB program package.^44^ Phylogenetic trees were calculated in ARB. QIIME 1.9.1^45^ was employed to group sequences into operational taxonomic units (OTUs) using 96%, 97%, and 94% nucleotide sequence similarity for archaeal, bacterial, and comammox *amoA*, respectively, to achieve approximately strain-level resolution, i.e. distinct AmoA amino acid sequences. See Supplementary Materials and Methods for detailed descriptions of PCR conditions, cloning procedures, and phylogenetic analyses.

### 16S rRNA gene amplicon library preparation and sequencing

PCR amplifications, library preparations, and DNA sequencing were conducted at the Environmental Sample Preparation and Sequencing Facility at Argonne National Laboratory following Earth Microbiome Project protocols^46^ using primers 515F^47^ and 806R^48^ as described in detail in the Supplementary Materials and Methods. Sequencing was performed on an Illumina MiSeq instrument using 150 cycle MiSeq Reagent kit v3 (Illumina, San Diego, Ca). Barcode removal, quality filtering, trimming, read merging, and chimera screening were conducted using DADA2^49^ implemented in the QIIME 2 package.^50^ Amplicon sequence variants (ASVs) were taxonomically classified using qiime feature-classifier and sklearn algorithm against the Silva database version 132.^51^ For detailed phylogenetic analysis ASV sequences were imported into ARB and manually aligned. Phylogenetic trees were calculated in ARB. Diversity metrics were determined using core-metrics-phylogenetic package implemented in QIIME2. Principal coordinate analysis (PCoA), canonical correspondence analysis (CCA), and distance-based redundancy analysis (dbRDA) were carried out using the vegan package 2.5–6^52^ in R version 3.6.3 to interrogate connections between microbial community structure and environmental variables (see Supplementary Materials and Methods for more details on amplicon sequencing and bioinformatic analyses).

### Quantification of *amoA* gene abundance by quantitative PCR

Ammonia monooxygenase subunit A *(amoA)* genes of archaeal, betaproteobacterial, and comammox *Nitrospira* were quantified by quantitative PCR (qPCR) using primers for archaeal *amoA,*^41^ bacterial *amoA,*^42^ and comammox *amoA* clade A and B^20^ using the QuantiTect SYBR^®^ Green qPCR kit (Qiagen, Germantown, MD) in a Bio-Rad IQ5 real-time PCR system (Bio-Rad, Hercules, CA). Genomic DNA of *Nitrososphaera viennensis* strain EN76 and *Nitrosospira briensis* Nsp10 was used as qPCR standards for archaeal and betaproteobacterial *amoA*, respectively. A comammox *amoA* standard was made from a comammox *amoA* clone plasmid carrying the most abundant sequence type found in our samples (OTU11). Further details of qPCR conditions and data analysis were provided in the Supplementary Materials and Methods. PCR amplification efficiencies were 86% for archaeal *amoA*, 90% for bacterial *amoA* and 96% for comammox *amoA*.

## Statistical analyses and data visualization

All Pearson correlations, ANOVA comparisons of means and post hoc tests were conducted using the respective functions in R version 3.6.3.^53^ If not otherwise noted data were deemed significant at *P* < 0.05. Phylogenetic trees were calculated and exported from ARB. All other data visualization was performed in R using ggplot2.^54^

## Results

### Nitrification with low N_2_O yields is closely linked to C-mineralization in EAA soils

**Fig. 1:**
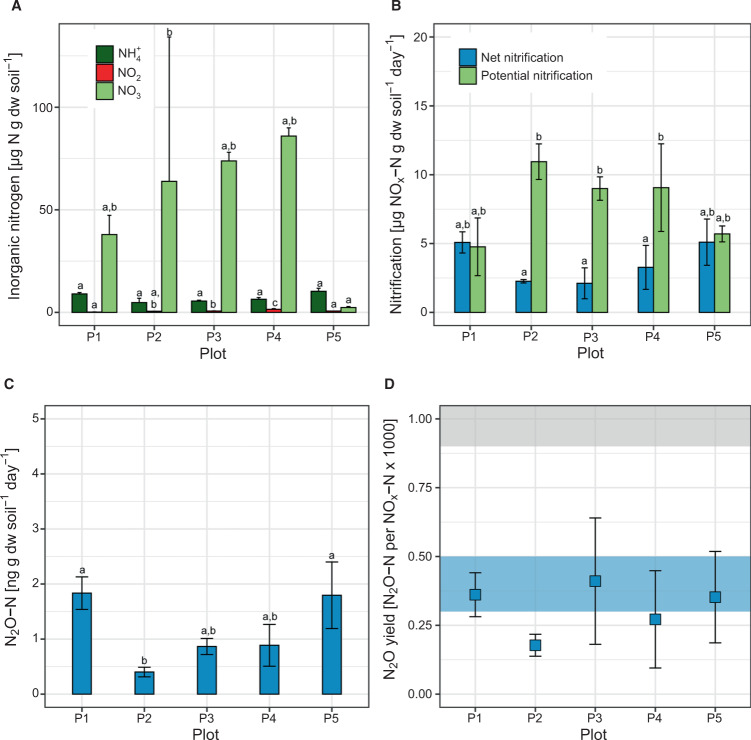
Inorganic nitrogen concentrations, nitrification rates, N_2_O production, and N_2_O yield in EAA soils. **A** Inorganic nitrogen concentrations, **B** Net nitrification (blue bars) and nitrification potentials (green bars) of EAA soils determined in microcosms and soil slurries, respectively. NO_x_–N refers to combined NO_2_^−^–N plus NO_3_^−^–N. **C** N_2_O–N production rates in net nitrification incubations. **D** Relative N_2_O yield of nitrification in net nitrification microcosms. Error bars denote standard deviations of *n* = 9 samples or incubations per plot. Different letters indicate significant difference (ANOVA with Tukey HSD test at α = 0.05 level). NO_3_^−^ concentrations in plot 2 varied significantly between 68.9, 63.9, and 215.5 µg g dw soil^−1^ in plot 2A, 2B, and 2C, respectively. The unusually high NO_3_^−^ concentration in plot 2C was confirmed twice. Shaded areas in (**D**) denote ranges of reported N_2_O yields of AOA (blue) and AOB (gray) in soils determined by selective AOB inhibition by 1-octyne (after Hink et al.^31^)

Net nitrification rates ranged from 2.11 ± 1.12 µg NO_x_–N (combined NO_2_^−^–N and NO_3_^−^N, plot 3) to 5.10 ± 1.68 µg NO_x_–N g^−1^ dry weight (dw) soil day^−1^ (plot 5). Although rates tended to be higher in plant-covered plots than in fallow plots, differences were not statistically significant (ANOVA with Tukey HSD test, *P* > 0.05, Fig. 1B). Notably, these rates were between 2 and 7 times higher than in e.g. unfertilized cropped soils in Oregon and Utah.^29,55^ Rates and N_2_O production in 0.1% acetylene-treated controls were assessed in preliminary experiments with samples collected in October 2017 and were below detection limit (data not shown). Nitrification potentials simultaneously determined in soil slurries tended to be higher in the fallow plots than in plant-covered plots with highest and lowest potentials in plot 2 (10.95. ± 1.29 µg NO_x_–N g^−1^ dw soil day^−1^) and plot 1 and 5 (4.76 ± 2.10 and 5.70 ± 0.58 µg NO_x_–N g^−1^ dw soil day^−1^) respectively. However the differences were not statistically significant. Nitrification potentials were significantly higher than net nitrification only in the fallow plots (Fig. 1B) and in a range comparable to other agricultural soils [e.g.^29,56,57^]. Growth in nitrification potential incubations was negligible within the first 48 h of incubations and NO_x_–N accumulated linearly (Supplementary Fig. 2). Unexpectedly, net nitrification and nitrification potential rates were indistinguishable in plots 1 and 5, suggesting that soil organic matter mineralization supported nitrification at maximum nitrification potential in these plots, and between 7.7 and 27.7% of nitrification potential in the fallow plots 2–4.

Cumulative N_2_O production and N_2_O yield followed a similar trend as net nitrification rates. Overall, N_2_O production ranged from 0.40 ± 0.09 ng N_2_O–N g^−1^ dw soil day^−1^ (plot 2) to 1.83 ± 0.30 ng N_2_O–N g^−1^ dw soil day^−1^ (plot 1) (Fig. 1C). The fallow plots also exhibited slightly lower N_2_O production rates than soils from plant-covered plots. N_2_O yields fell into a rather narrow range between 0.18 ± 0.04 ng N_2_O–N per µg NO_x_–N (plot 2) and 0.41 ± 0.22 ng N_2_O–N per µg NO_x_–N (plot 3), averaging 0.31 ± 0.09 ng N_2_O–N per µg NO_x_–N between all plots (Fig. 1D). Similar N_2_O yields between 0.3 and 0.6 ng N_2_O–N per µg NO_x_–N were recently reported for AOA activity in soils treated with 1-octyne to inhibit AOB.^31,32^ The low N_2_O yields contrasted reported N_2_O yields of AOB of >0.9 ng N_2_O–N per µg NO_x_–N and suggested that AOA or comammox bacteria were the main drivers of nitrification in EAA soils.

Carbon mineralization rates ranged from 8.81 ± 0.94 (plot 4) to 25.14 ± 1.16 µg C g^−1^ dw soil day^−1^ (plot 1) (Supplementary Fig. 2). Again, plant-covered plots 1 and 5 showed the higher activities, whereas plots 2 and 4 with the longest fallow periods showed the lowest activities. Carbon mineralization rates were significantly different in plots 1, 3, and 5 (*P* < 0.01), but not plot 2 and 4.

**Fig. 2:**
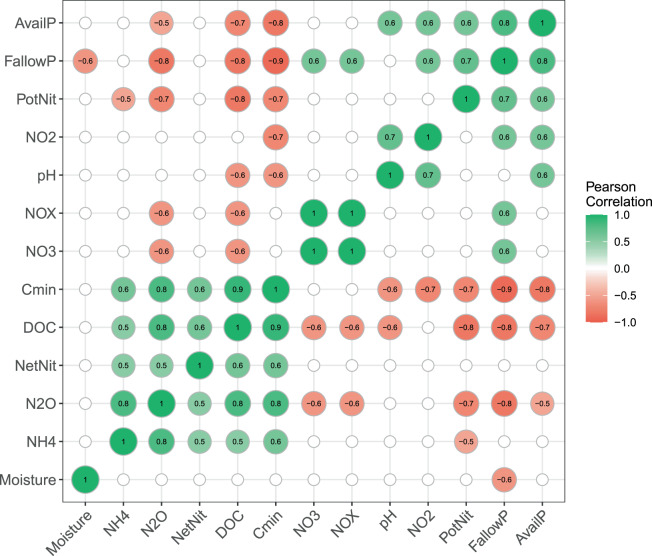
Pearson correlation coefficients of soil edaphic factors and microbial activity measurements. Only significant correlations (*P* < 0.05) are shown (*n* = 15). NH4 [NH_4_^+^], N2O N_2_O production rate, NetNit Net nitrification rate, DOC dissolved organic carbon, Cmin Carbon mineralization, NO3 [NO_3_^−^], NOX [NO_2_^−^] + [NO_3_^−^], NO2 [NO_2_^−^], PotNit Nitrification potential rate, FallowP Fallow period, AvailP Available phosphorous.

Pearson correlation coefficients of environmental factors and microbial activities showed strong positive correlations of DOC, C-mineralization, N_2_O production, and to a lesser degree net nitrification, implying linked microbial C- and N-mineralization in these highly organic soils (see Supplementary Results for more detailed discussion of soil C-, N- and P chemistry). DOC concentrations were also significantly negatively correlated with fallow period, total NOx, and available P (Fig. 2). Notably, fallow period showed the largest number significant correlations with strong negative associations to DOC, C-mineralization rate, N_2_O production rate, and positive correlations with NO_2_^−^ and NO_3_^−^ concentrations, available P, and nitrification potential (Fig. 2). Together these results suggested that microbial degradation of soil DOC and nitrification were closely linked and that fallow periods significantly contributed to decline of DOC and net nitrification and build-up of nitrification potential.

### Ammonia-oxidizer communities are highly AOA dominated

**Fig. 3:**
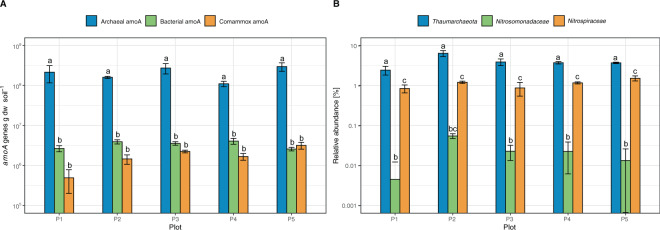
Abundance of archaeal, canonical bacterial, comammox, and canonical nitrite-oxidzing bacteria in EAA soils based on *amoA* genes and relative 16S rRNA gene frequencies. **A** Abundance of archaeal, canonical bacterial and comammox *amoA* genes in EAA soil plots 1 through 5 determined by qPCR. Error bars represent standard deviations of three sub-plots with three replicates each (*n* = 9). **B** Relative abundance of *Thaumarchaeota, Nitrosomonadaceae*, and *Nitrospiraceae* clades based on relative frequency of 16S rRNA genes in amplicon sequence datasets. Error bars represent standard deviations between three sub-plots (*n* = 3). Different letters indicates significant difference within plot. Archaeal *amoA* gene copy numbers were significant higher than bacterial and comammox *amoA*, differences between bacterial and comammox *amoA* were not statistically significant (ANOVA with Tukey HSD test at α = 0.05 level). *Thaumarchaeota* were significantly more frequent than *Nitrosomonadaceae* and *Nitrospiraceae* in all plots, *Nitrospiraceae* were significantly more frequent than *Nitrosomonadacea*, except in plot 2 (ANOVA with Tukey HSD test at α = 0.05 level).

Archaeal *amoA* genes ranged between 1.10 × 10^8^ ± 1.66 × 10^7^ (plot 4) and 2.97 × 10^8^ ± 7.13 × 10^7^ (plot 5) g^−1^ dw soil and were not significantly different between plots (Fig. 3A). AOB and comammox clade A *amoA* genes were 27- to 120-times and 65 to 430-times less abundant than AOA *amoA* genes, respectively, ranging from 2.58 × 10^6^ ± 2.59 × 10^5^ to 4.08 × 10^6^ ± 6.83 × 10^5^ g^−1^ dw soil (AOB) and 4.94 × 10^5^ ± 2.93 × 10^5^ to 3.18 × 10^6^ ± 6.20 × 10^5^ g^−1^ dw soil (comammox clade A). Comammox clade B *amoA* genes were not detected in any of the plots by endpoint PCR or qPCR. Overall, neither AOA, AOB, nor comammox gene copy numbers varied significantly between plots (*P* > 0.05, Fig. 3A). Assuming between 2 and 3 *amoA* gene copies per AOB genome^58^ and 1 copy per AOA and comammox *Nitrospira* genome, these results suggest that AOA were between 54- and 430-times more abundant than AOB and comammox *Nitrospira* in our soils.

The numerical dominance of AOA determined by qPCR was corroborated by the relative frequencies of nitrifiers in 16S rRNA gene amplicon data (Fig. 3B). *Thaumarchaeota*, principally AOA, as no Group 1.1C-associated sequences were found (Supplementary Fig. 4), constituted between 2.42 ± 0.60% (plot 1) and 6.37 ± 1.08% (plot 2) of all 16S rRNA sequences, with intermediate frequencies of 3.87 ± 0.73%, 3.70 ± 0.25% and 3.68 ± 0.16% in plot 3, plot 4, and plot 5, respectively. AOB genera *Nitrosomonas* sp. and *Nitrosospira* sp. (designated as *Nitrosomonadaceae* in Fig. 3B) were rare, ranging between 0.006% in plot 1 and 0.055% in plot 2, 116- and 537-times less frequent than *Thaumarchaeota*. As common for agricultural soils, all identified canonical nitrite-oxidizing bacteria (NOB) were affiliated with *Nitrospiraceae* and ranged from 0.80 ± 0.30% (Plot 3) to 1.33 ± 0.17% (Plot 5) of all 16S rRNA gene reads. Only 1 out of 37 *Nitrospira* sp.-affiliated ASV clustered closely with known comammox bacteria (Supplementary Fig. 5). This ASV was detected with increasing relative frequency of 0.025 ± 0.024% in plot 3 to 0.116 ± 0.006% of all 16S rRNA gene reads, between 32- and 152-fold lower than *Thaumarchaeota-*affiliated AOA. The predominant *Nitrospira* sp.-affiliated ASVs clustered with *N. japonica*, *N. moscoviensis*, and *N. lenta*, although sequences of all six main *Nitrospira* lineages were detected. Together the low comammox *amoA* copy numbers and low relative frequency of 16S rRNA genes indicated low prevalence of comammox in EAA soils (Fig. 3).

**Fig. 4:**
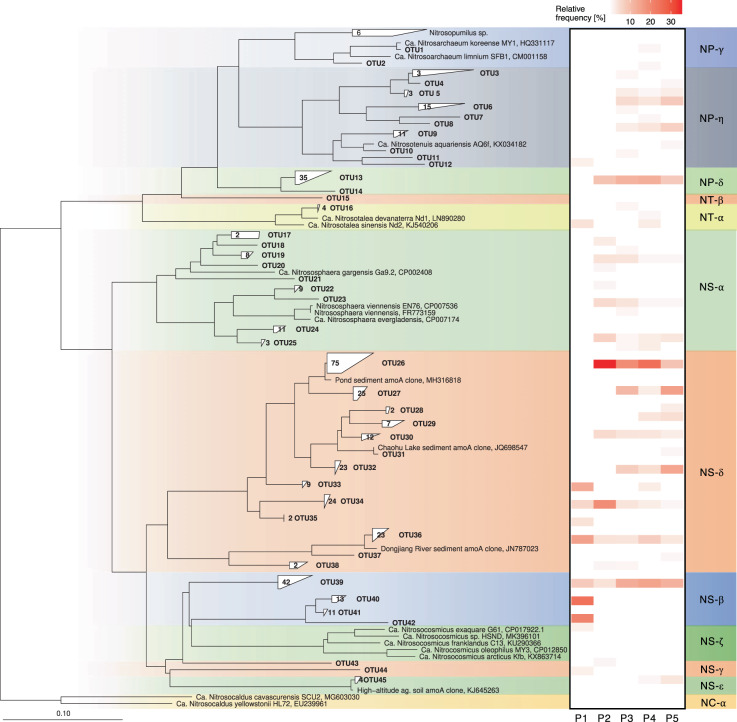
Neighbor-joining phylogenetic tree of archaeal *amoA* gene sequences retrieved from EAA plots 1–5 and heat map of relative abundance within each plot (in %). Tree was calculated based on 595 aligned positions using neighbor-joining algorithm with Jukes–Cantor correction. Sequences with more than 96% sequence similarity on nucleotide level were grouped into single OTUs. The total number of sequences within a given OTU, if more than one, is indicated in each tree leaf.

Analysis of *amoA* and 16S rRNA genes revealed a diverse AOA community, contrasting low diversity AOB and comammox communities. A total of 396 archaeal *amoA* sequences clustered into 45 OTUs with a 96% sequence similarity, phylogenetically affiliated with 10 distinct AOA lineages (Fig. 4). The largest number of archaeal *amoA* sequences fell into the NS-δ group (uncultivated *Nitrososphaera*-sister group, OTU26–38) which accounted for between 37.7% (plot 1) and 64.6% (plot 2) of all archaeal *amoA* sequences, and ~50% of all sequences in plot 3–5. This cluster with most closely related environmental sequences from ponds, lake sediments, and rivers was approximately equidistant (70–80% nucleotide sequence similarity) from cultivated *Nitrosocosmicus* sp. and *Nitrososphaera* sp. in NS-ζ and NS-α lineages, respectively. Interestingly, the most abundant OTU (NS-δ OTU26) was found only in agriculturally managed plots and increased with increasing fallow period from 8.7% in plot 5 to 25.0% and 35.4% in plot 4 and 2, respectively, suggesting significant growth of this ecotype during the fallow period. In contrast, the more acidic unmanaged plot 1 was dominated by two OTUs in the NS-β lineage, together accounting for 45.3%. Only 4 OTUs found in plot 1 were found in at least one other plot, but were not numerically abundant in those plots. Overall diversity was similar in plot 3–5 (21–23 OTUs) and somewhat lower in plot 1 and 2 (11 and 15 OTUs, respectively).

Similar overall *Thaumarchaeota* patterns were observed in 16S rRNA gene datasets with smaller fractions associated closely with cultivated AOA lineages NP-γ (*Nitrosarchaeum* sp.), NP-η (*Nitrosotenuis* sp.), NT-α (*Nitrosotalea* sp.), NS-α (*Nitrososphaera* sp.), and NS-ζ (*Nitrosocosmicus* sp.). Again, the larger proportion of sequences of the managed plots associated with 12 ASV’s clustering with environmental sequences of the NS-δ lineage, equally distant between the *Nitrososphaera* and *Nitrosocosmicus* lineages, together accounting for between 20.6% in plot 1 and 66.3% in plot 2, and between 50.3% and 59.5% in plot 3 -5 (Supplementary Fig. 4). Similar to the archaeal *amoA* dataset, one single ASV (ASV11) dominated the amplicon dataset, constituting between 34.7 and 39.5% of AOA-related sequences in the agriculturally managed plots 2–5, and below 1% in plot 1. However, fewer *Nitrosocosmicus*-associated sequences were found in the 16S rRNA amplicon dataset than in the *amoA* dataset. The most striking difference between both datasets were observed in plot 1, where NS-β-related sequences were frequent in the *amoA* dataset, but represented only a minor fraction in the 16S rRNA gene amplicon data. Instead, a distantly *Nitrososphaera*-related NS-α phylotype with 69.7% frequency vastly dominated the plot 1 dataset.

**Fig. 5:**
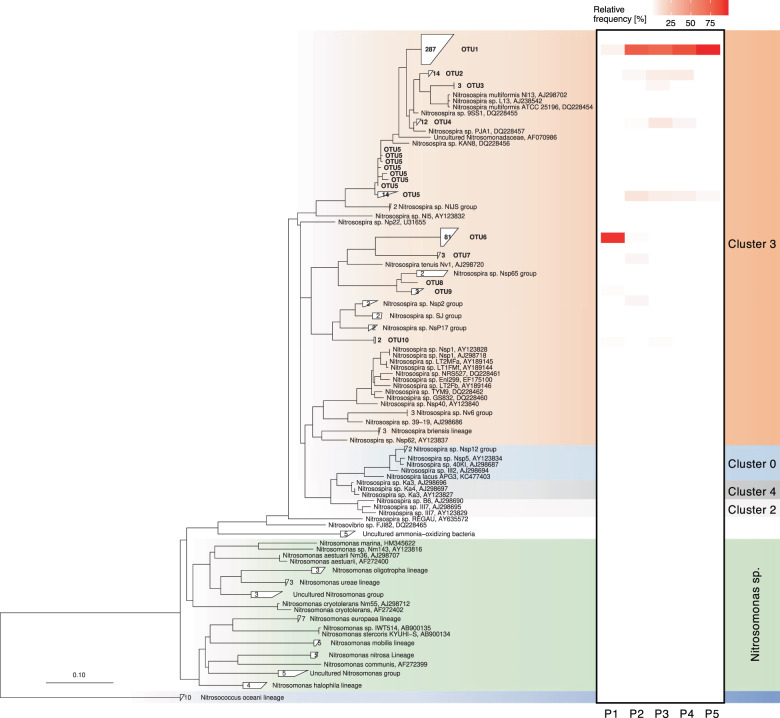
Neighbor-joining phylogenetic tree of canonical bacterial *amoA* gene sequences retrieved from EAA plots 1 through 5 and heat map of relative abundance within each plot (in %). Tree was calculated based on 453 aligned positions using neighbor-joining algorithm with Jukes–Cantor correction. Sequences with more than 97% sequence similarity on nucleotide level were grouped into single OTUs. The total number of sequences within a given OTU, if more than one, is indicated in each tree leaf. Labels on the right refer to *Nitrosospira* sp. sequence clusters 0–4.

AOB communities shifted from *N. multiformis*-related OTUs dominating in the managed plots 2–5 to *N. tenuis*-related OTUs in native plot 1. A total of 427 bacterial *amoA* clone library sequences from plots 1–5 were assigned to ten OTUs based on 97% DNA sequence similarity and affiliated with *Nitrosospira* cluster 3 (Fig. 5). Managed and native plots were dominated by a single OTU comprising between 78.3 and 97.5% of sequences (OTU1) in managed plots and 93.0% (OTU6, 80 of total 86 clones) in native plot 1, respectively. Only 4–7 OTUs were observed in each plot. No *Nitrosomonas sp*. were found in *amoA* clone libraries. Although both, *Nitrosospira*- and *Nitrosomonas*-related sequences were detected in the 16S rRNA gene amplicon dataset, the total number of sequences was too low to reliably estimate relative frequency of AOB (data not shown).

**Fig. 6:**
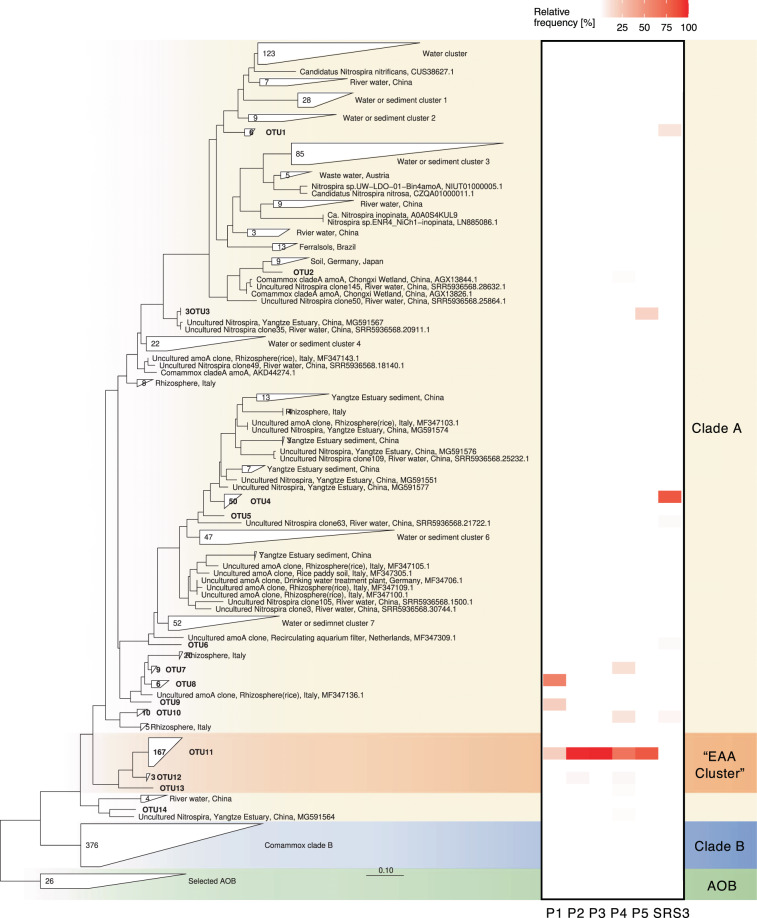
Maximum-likelihood phylogenetic tree of comammox *Nitrospira* sp. *amoA* gene sequences retrieved from EAA soil plots 1 through 5, and Everglades wetland soil SRS3. The tree was calculated using RAxML algorithm with GTRGAMMA-25 rate distribution model and rapid hill climbing algorithm based on 383 nucleotide positions. Heat map shows relative abundance of each OTU within each soil (in %). Sequences with more than 94% sequence similarity on nucleotide level were grouped into single OTUs. The total number of sequences within a given OTU, if more than one, is indicated in each tree leaf. Labels on the right refer to comammox *amoA* clade A and B.

A total of 259 comammox *amoA* clone library sequences were obtained from all five plots and fell into 14 distinct OTUs with 94% nucleotide sequence identify cutoff within comammox *amoA* clade A. Most sequences affiliated with a single OTU within a cluster of novel sequences (“EAA Cluster”, Fig. 6), with most closely related sequences retrieved from soil rhizosphere. Overall diversity was low, with only 1–6 OTUs in total. For comparison, we also analyzed comammox clade A diversity in a reference site within the Everglades wetland marshes (SRS3), resembling the EAA soils prior to drainage and cultivation. Sequences from SRS3 were also dominated by a single OTU (OTU4) with 83% affiliated sequences and closely related to sequences retrieved from the Yangtze River Estuary, China, and were distinct from the predominant EAA soil OTUs (Fig. 6). Cloning efficiency of comammox *amoA* was notably low between 5.2% (plot 1) and 82.3% (plot 4) positive clones, respectively, with intermediate efficiencies of 33.3%, 68.8%, and 17.7% from plot 2, 3, and 5, corroborating other recent observations of a rather broad inclusiveness of the employed primer set and significant off target amplification, strongly indicating that comammox *amoA*, despite its low abundances, were likely still overestimated.^20^

We further examined the 16S rRNA gene amplicon dataset for NOB and putative comammox *Nitrospira* sp. (Supplementary Fig. 5). All NOB found in the dataset affiliated with the six lineages within genus *Nitrospira*, ranging from 0.84% (plot 1) to 1.34% relative frequency (plot 5). Among *Nitrospira*-affiliated sequences the predominant ASV5 clustered with *N. japonica* (99.6% sequence similarity), decreasing in relative frequency from 84.5% (plot 1) to between 40.8 % (plot 3) and 50.7% (plot 2). The second most abundant ASV13 affiliated closely with *N. moscoviensis* (99.6% sequence similarity) and ranged between 12.4% (plot 2) and 23.0% (plot 4) and was absent in plot 1. Only ASV35 affiliated closely with known comammox *Nitrospira (96.5% sequence similarity to Ca*. Nitrospira nitrosa). This ASV was absent from plot 1 and 2, and increased from 2.6% to 8.8% of all *Nitrospira*-affiliated sequences from plot 2–5, equivalent to 0.025%–0.116% of all 16S rRNA gene sequences. Comammox-affiliated sequences were 65- to 430-times (*amoA*) and 32- to 152-fold (16S rRNA gene relative frequency) less abundant than AOA.

Together, the results indicate a low comammox diversity in both EAA soils and Everglades wetlands with rhizosphere-associated clades dominating in EAA soils, and distinct river and estuary-associated sequences dominating in the wetlands. Although, the clone library datasets were obviously lacking in sequencing depth, the overall congruence of *amoA* and 16S rRNA gene data implied that AOA phylotypes were generally identified consistently with both gene targets, and that both managed and unmanaged plots were dominated by a diverse assemblage of AOA phylotypes, accompanied by a very limited diversity of AOB and comammox *Nitrospira* of much lower abundance. Some taxonomic bias still exists in one or both of these approaches that warrants further investigation.

### Effects of pH and soil moisture on overall and archaeal nitrifier community structure

**Fig. 7:**
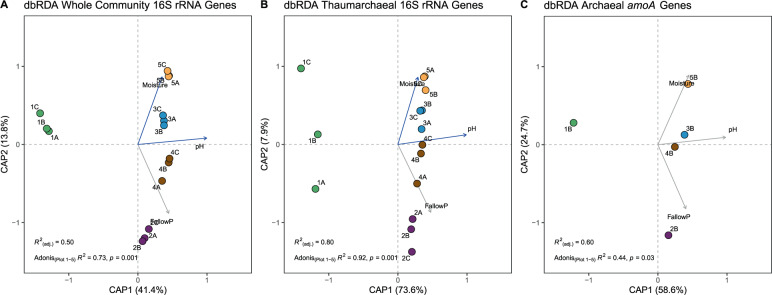
Distance-based redundancy analysis (dbRDA) of EAA microbial communities and soil edaphic factors. Shown are dbRDA analyses based on whole microbial community 16S rRNA genes (**A**), thaumarchaeal 16S rRNA genes (**B**), and archaeal *amoA* genes (**C**).Proportions explained by the first two dimensions of each ordination are shown in parentheses. Overall significance Q6 (R2) and significance of differences between plots based on Adonis test are shown. A two-factor model with pH and moisture was sufficient to explain most of the variability between plots 1–5, with three-factor models not significantly improving the overall fit (pairwise ANOVA between two and three-factor models). Comparison of p values of two- and three-factor models with all soil edaphic factors and microbial activities tested revealed the model with pH and fallow period with only slightly lower overall fit. See Supplementary Fig. 7 for corresponding PCoA and CCA analyses.

We further investigated whether the observed correlations of DOC, C-mineralization, N_2_O production, and to a lesser degree net nitrification influenced archaeal nitrifier and overall microbial community structure. Surprisingly, overall microbial α- and β-diversity differed significantly between plots. Shannon indexes, observed OTU’s, and Faith’s phylogenetic diversity all indicated lower α-diversity in plant-covered plots 1 and 5, and higher diversity in fallow plots 2–4 (*P* < 0.05, Supplementary Fig. 6). Notably, distance-based RDA (dbRDA) showed clustering of samples by plots primarily along gradients of pH and moisture in whole microbial communities, as well as in thaumarchaeal communities by 16S rRNA and *amoA* gene sequences (Fig. 7 and Supplementary Fig. 7). A two-factor model fit with pH and moisture gave the best fits in dbRDA and CCA. However, models with pH and fallow period as explanatory variables resulted in only a slightly lower goodness of fit. PCoA, dbRDA, and CCA recovered overall similar patterns, with only plot 3 and 4 samples partially overlapping in PCoA and CCA (Fig. 7, Supplementary Fig. 7A, B, D, E), indicating that dbRDA and CCA did not impose significant distortions on the overall ordinations. These results imply that the whole microbial communities and thaumarchaeal nitrifier communities were both structured similarly by pH and soil moisture, and that other edaphic factors (DOC, NH_4_^+^, NO_2_^−^, NO_3_^−^, available P) and microbial activities (C-mineralization, net nitrification, nitrification potential, and N_2_O production) were not correlated to soil microbial community structure.

## Discussion

The discovery of AOA and comammox bacteria have drastically expanded the diversity of nitrifiers.^21,25^ Still little is known about the controls on contribution of AOA, AOB, and comammox to nitrification activity in terrestrial systems, and no single approach can currently distinguish between activities of all three groups. 1-octyne has been used as selective inhibitor of AOB activity and was shown to have little effect on AOA activity.^29,30,32^ However, it is not yet known whether comammox bacteria with a presumably more AOB-like AMO enzyme structure are also inhibited. On the other hand, 2-phenyl-4, 4, 5, 5,-tetramethylimidazoline-1-oxyl 3-oxide (PTIO) was shown to selectively inhibit AOA and comammox activity.^23,40,59,60^ But the reactivity of its nitrogen free radical makes it unsuitable for soil studies. Recent demonstration that AOA activity (in the presence of 1-octyne) in soils was associated with low N_2_O yields of 0.03–0.05‰ and activity of AOB with N_2_O yields of ≥0.9‰^31,32^ indicated the potential of N_2_O yield mixing ratio as a tool to estimate the contribution of AOA and AOB to nitrification.^18,31^ This would be particularly attractive as it would neither require altering substrate concentrations, nor application of inhibitors that could potentially stimulate one or the other group. N_2_O yield of net nitrification in our study was consistently in the low yield range of AOA activity, indicating that AOA-dominated nitrification activity in EAA soils, and that N_2_O yields of 0.2–0.5 ng N_2_O–N per µg NO_x_–N also hold up in situ in the absence of inhibitors (Fig. 1D). Notably, laboratory cultures of *N. viennensis* and *N. inopinata* showed slightly higher N_2_O yields (~0.7‰) than AOA in bulk soils (Fig. 1D).^23,31,32,61^ Our results corroborate the low N_2_O yields of AOA in soils determined in the presence of 1-octyne and that substrate saturation in laboratory cultures may somewhat increase the overall N_2_O yield.^61^ Furthermore, these results support the utility of agricultural management practices that selectively limit AOB activity to curb N_2_O emissions from agricultural systems.^18,19^

N_2_O yield can likely not distinguish AOA and comammox activity. However, two lines of evidence indicate that comammox activity was insignificant in EAA soils: Comammox *amoA* genes and potential comammox-affiliated ASV’s in the 16S rRNA gene amplicon dataset were 65- to 430-times (*amoA*) and 32- to 152-fold (16S rRNA gene relative frequency) less abundant than AOA (Fig. 3A, Supplementary Fig. 5). Based on the qPCR values and uniform activity among all comammox bacteria, these cells would need to oxidize between 6.7 (plot 3) and 73.4 fmol ammonia cell^−1^ day^−1^ (plot 1) in order to contribute just 10% of the observed net nitrification rate. Such a rate would be near or above the maximum activity of *N. inopinata* (15–40 fmol cell^−1^ day^−1^ under optimal growth conditions at substrate saturation (estimated from Fig. 2d in.^11^) Affinity calculations by Kits et al.^24^ also showed that, albeit having a lower half saturation constant for ammonia, *N. inopinata* exhibited a lower specific affinity (i.e. actual oxidation rate per unit biomass at [total NH_3_ + NH_4_^+^] → 0) than some tested AOA.^24^ Although the trade-offs between specific substrate affinity and molar growth yield of comammox in long-term competition with AOA remain unknown, and we cannot rule out that other more efficient and faster growing comammox bacteria may exist in agricultural soils, our results collectively indicate that AOA appeared to be responsible for the bulk of nitrification activity and N_2_O production in EAA soils. Further research is needed to substantiate the ecological roles of the low diversity comammox assemblage in these soils.

Correlations of abundance and diversity of AOA with environmental factors including pH, moisture, temperature, NH_4_^+^, and organic matter have been investigated intensively, with pH, temperature, and NH_4_^+^ often emerging as important factors in upland soils (reviewed in ^14,15,27,62,63^). Our analyses were primarily targeting microbial activities and therefore had limited statistical power to interrogate influence of environmental factors on microbial communities. Within our five plots, soil pH and moisture content were the only significant environmental factors potentially governing AOA, as well as overall microbial community structure in EAA soils. Indeed, these soils undergo a wet summer and dry winter seasonal cycle. Moisture was negatively correlated with fallow period, potentially a reflection of the expected vulnerability of fallow soils to flooding during the rainy summer season and dry-out during the late fall and winter season. Net nitrification activity was correlated with dissolved organic carbon concentration, carbon mineralization, and soil ammonium concentrations (Fig. 2). Together, these results suggest that AOA communities and nitrification activity in EAA soils were likely not directly tied to specific plants or root exudates, but support an indirect link between plant and nitrifier activities through pH, moisture, or stimulation by plants of the DOC pool, soil organic matter mineralization and nitrification. Based on our study we cannot yet distinguish whether this may happen directly through plant priming as previously proposed,^15,27,33,34^ or indirectly through regulation of soil moisture that in turn could affect which microbes were active in the soil. For example, it is conceivable that moisture could primarily influence other microbial guilds in the soil (e.g. N-mineralizing microbes), which in turn could influence AOA communities by the types and quantities of N substrates that become available for nitrifiers (e.g. ammonia, urea, or cyanate). Further experiments including e.g. adjustments of soil moisture contents or collection of seasonal data would be needed to further deconvolute the controlling factors and functional interactions within the soil microbiome that may govern nitrification rates over time.

The role that plant communities play in directly or indirectly regulating soil nitrification and nitrifier communities e.g. via competition for N source or biological nitrification inhibition still remain poorly understood.^64,65^ Nonetheless, it is generally assumed that plant nitrogen assimilation controls reactive nitrogen pools in soils and strongly dominates over microbial nitrification particularly in high yield agricultural systems.^18,65–68^ Similar nitrifier abundances and nitrification rates in EAA plots with shared multi-year legacy of sugar cane cultivation, followed by either continued sugar cane cultivation (plot 5) or variations of fallow treatment (plot 2–4), suggest that plants and soil AOA may not compete for reduced nitrogen in these soils. Direct determination of nitrogen flux rates in intact soils is technically extremely challenging. This is particularly true, if potentially high-affinity microbial processes are involved since steady-state activities are often sensitive to disturbances, as has been shown e.g. for marine microbes, including archaeal nitrifiers.^69,70^ We did not directly determine form and rate of plant nitrogen uptake here, and net nitrification rates in microcosms may not exactly reflect in situ nitrification rates. However, low NH_4_^+^ concentrations, similar nitrifier abundances, and steady net nitrification rates in plant-covered and fallow plots, with concurrent high soil NO_3_^−^ accumulation only in fallow plots, indicated that native plant assemblage in plot 1 and sugar cane in plot 5 must have drawn significantly on NO_3_^−^ produced by AOA and NOB as a primary nitrogen source (Fig. 1A, B). Indeed, the determined NO_3_^−^ concentrations in the fallow soils were equivalent of accumulated net nitrification activity of 26–35 days, a quite realistic value 6 to 8 weeks after the beginning of the dry season and declining precipitation, and associated nitrogen leaching in the subtropical region of South Florida. In contrast, the NO_3_^−^ levels in the plant-covered plots reflect less than 1 day (sugar cane, plot 5) and ~7 days nitrification activity (native, plot 1), suggesting tightly linked nitrification and plant nitrogen uptake, especially in the densely plant-covered cropped soil.

Intriguingly, nitrification potentials and net nitrification were similar in plant-covered plots. Although we did not examine active nitrifier populations in both incubation types, and we cannot exclude that both assays captured activity of different ammonia oxidizer populations, the similarity of rates between net nitrification incubations and nitrification potential incubations using growth media instead of phosphate buffer may suggest that AOA activity was at or near maximum capacity. This is contrasting less fertile agricultural soils with history of inorganic fertilizer application that can display more than 10-fold difference between net- and potential nitrification rates [e.g. ^28,55,57^]. In contrast, the higher nitrification potentials in fallow soils (plots 2–4) indicate the possibility of some build-up of nitrification potential and AOA growth over time during the fallow period, albeit, not significant enough to be detectable by qPCR and relative abundances of 16S rRNA genes (Figs. 1 and 3). If more significant growth of AOA would have been possible, as we initially hypothesized, it should have become apparent in the *amoA* qPCR assays in the shorter (plot 3) or longer fallow treatments (plot 2 and 4). Alternatively, if AOA and plants competed for reduced nitrogen, or AOA were inhibited by plant root exudates, population size and activity should have been significantly lower in plots with more than 2 years of continuous high yield sugar cane production, than in neighboring fallow plots after several month of fallow period and NO_3_^−^ accumulation. However, further research is needed to better characterize the diversity of substrates that can fuel nitrification in these soils.

Comparison of sugar cane N assimilation and nitrification rates based our measurements suggest that in plot 5 nitrification could suffice to meet the cane N demand throughout the entire growth season. Sugar cane N content and uptake have been characterized in detail. Given N content of 4.5–6.5 g N per kg sugar cane and a stem-to-root biomass ratio of 1:2,^71^ the December 2017 cane harvest in plot 5 of 68.4 ton per ha (EREC farm manager, pers. communication) would be equivalent to 1.03 ton ha^−1^ cumulative N assimilated by sugar cane throughout the 2017 growth season. For comparison, using the net nitrification rate in plot 5 determined at daily average soil temperature of 28 °C in the cooler December month (Fig. 1B) as a base line rate throughout the season, a soil depth of 30 cm,^36^ and bulk density of EAA soils of 350 kg m^−3^, net nitrification in plot 5 would yield ~1.59 tons of NO_3_^−^–N ha^−1^ during the same 10-month growth period. Given the seasonality of soil temperatures, temperature sensitivity of microbial mineralization activity with Q10 values of 2.5 for tropical systems^72^ and soil AOA activity with optimal activities near 40 °C,^30^ these rate estimates for the winter season likely represent conservative estimates for the overall growth cycle. Although crop N uptake is not steady and peaks particularly during mid-season shoot growth, and nitrogen leaching may be significant during these wet summer month in the EAA, the estimated net nitrification rate would be well in the range to supply sufficient N throughout the growth season for a predominantly nitrification-derived, NO_3_^−^-based sugar cane growth.

Soils with sufficiently high organic matter content to supply N primarily or exclusively through microbial mineralization with little or no N fertilizer application, like the EAA soils investigated here, are rare. However, many upland plants at neutral or slightly alkaline soil pH are known to readily use or even prefer NO_3_^−^ as a nitrogen source [e.g. ^66,68,73^]. To the best of our knowledge, no experimental demonstration of linked high-affinity nitrification by AOA and plant nitrogen uptake has been provided to date. This may at least in part be attributed to impact of inorganic nitrogen fertilizer application on nitrifier community structure, plant nitrogen uptake and standing stock biomass in productive agricultural systems.^2,8,15,19,27^ Although we cannot completely rule out historical differences in management of the EAA plots investigated here, our data strongly indicate that nitrification activity by AOA was an integral part of the nitrogen cycle in this soil, providing plants with a nitrogen source derived entirely from organic matter mineralization. More broadly, these results suggest that AOA could exert a more significant, and thus far overlooked role, in controlling the reduced nitrogen pool in terrestrial ecosystems, potentially contradicting the prevailing notion of nitrification solely as a detriment in agricultural systems and efforts to enhance plant nitrogen use efficiency through biological or chemical nitrification inhibition.^8,18,65^ It is intriguing to speculate that also in other less fertile soils, plants, e.g. under sub-optimal conditions, could benefit from AOA activity to enhance access to nitrogen in the more mobile form of NO_3_^−^, and that beneficial molecular interactions between plants and AOA may be more widespread than recently shown in a laboratory *Arabidopsis* system.^74^

## Conclusions

In summary, using highly fertile, well-aerated organic agricultural soils, we show that highly AOA-dominated nitrifier communities and nitrification activity are integral to the nitrogen cycle in these soils, irrespective of presence or absence of plant cover. Low N_2_O yields of AOA-dominated soil nitrification confirm previous inhibitor-based studies, indicating a significant potential for N_2_O emissions reduction through management practices that selectively reduce AOB activities. Our results suggest an unanticipated potential for interactions between microbial nitrogen mineralization, AOA activity, and plant nitrogen uptake in soil ecosystems.

## Supplementary information

Supplemental Materials, Methods and Results

Supplemental Figures 1–7

## Data Availability

The paired-end Illumina 16S rRNA sequences of this study have been deposited at the National Center for Biotechnology Information (NCBI) Sequence Read Archive (SRA) under project accession number of PRJNA691051. Archaeal, bacterial, and comammox *amoA* sequences have been deposited in NCBI Genbank under accession numbers MW461001–MW462044.
