# Supplementary material for: Ammonia-oxidizing archaea are integral to nitrogen cycling in a highly fertile agricultural soil
Source: ISME Commun. 2021 Jun 1;1:19. doi: 10.1038/s43705-021-00020-4 (PMC9723749; doi:10.1038/s43705-021-00020-4)
Supplement: Supplementary file 1 — Supplemental Materials, Methods and Results [file 43705_2021_20_MOESM1_ESM.pdf]

Contents of Supplemental Materials

Supplementary Materials and Methods Page 2

Supplementary Results Page 12

Supplementary Materials and Methods

Sample collection

Soil samples were collected in December 2017 at the University of Florida Everglades Research and Education Center located within in the Everglades Agricultural Area (EAA) near Belle Glade, FL (Supplementary Figure 1). The Everglades Agricultural Area (EAA) is an area of approximately 3,000 km^2 of farmland representing about 27% of total historical reach of the Everglades wetlands converted to agricultural land after drainage between 1900 and 1920 [75, 76]. The soils in the EAA are characterized as organic histosol with 75% to 85% organic matter content [77] and currently approximately 80% of the agricultural land is planted with sugarcane [78].

We selected four agriculturally managed plots in close proximity differing in crop cover and fallow period since December 2016 (approximately one year before sampling), following three years of continuous sugarcane cultivation (plot 2 - 5), and one unmanaged control plot (plot 1) (Supplementary Figure 1). Following sugarcane harvest in plot 2 - 5 in November 2016, plot 2 was planted with spinach from January to May 2017, followed by a 28-week fallow period before sampling in the first week of December 2017. Plot 3 was planted with sweet corn from January to May 2017, followed by flooding and rice cultivation from May until October 2017 and an 8-week fallow period from October to December 2017. On plot 4 sweet corn was grown from January to May 2017, also followed by a 28-week fallow period until sampling in December 2017. On plot 5 sugar cane was cultivated for a third consecutive year until harvest in November 2017,

one week before sampling. In the fallow plots, growth of weeds was suppressed by herbicide application and tilling. At the time of sampling all four managed plots were either fallow (Plot 2 to 4) or sugarcane harvested within one week prior to sampling (plot 5). The unmanaged control plot 1 was covered with a mixed plant community and was mowed once a year (last in April 2017) for wildfire prevention and neither fertilized, nor managed with pesticides for at least 30 years.

From each plot three composite bulk soil samples were collected between 50 and 200 m apart. Each composite sample consisted of 9 individual 10-cm topsoil cores collected within a 10 x 10 m square with 5cm diameter plexiglass corers (or sharp spade, in case plan roots had to be cut). The cores were combined, plant roots and rocks were removed, and the samples homogenized by sieving and mixing. Composite samples were divided and immediately frozen on dry ice for molecular analyses or stored on wet ice for nitrification potential, net nitrification and other soil analyses, respectively. Samples for soil nutrient analyses (5 g wet weight) were immediately suspended in 45 ml 2M KCl (adjusted to pH 6 with 1mM KH_2PO_4) and refrigerated until further processing after return to the laboratory.

Physical and chemical soil analyses

Soil temperature was recorded at the time of sampling in the field. Soil pH was determined using the soil slurry method. Briefly, ten grams of soil were suspended in 20 ml double deionized water (EMD Millipore Synergy UV) and homogenized by shaking for 1 hour at room temperature before recording pH. Water content was determined by

weight loss after drying 20 g fresh soil at 105°C for 24 hours in pre-weighed glass containers.

Soil nutrients (NH_4^+, NO_2^- and NO_3^-) were determined colorimetrically following extraction with 2 M KCl. Briefly, 5 g wet soil was extracted with 45 ml of 2 M KCl solution (adjusted to pH 6 with 1 mM KH_2PO_4 to improve uniformity of extraction and subsequent assays) by shaking for 1 hour at 50 rpm. Samples were centrifuged for 10 min at 3,000 *g* and 10 ml supernatant was filtered through a 0.45 µm Nylon filter. Samples were refrigerated until analysis within 1-5 days. NH_4^+ and NO_2^- were determined using the salicylate method [79] and sulfanilamide-NED method [80], respectively. NO_3^- was determined following aqueous vanadium reduction using the method by García-Robledo et al. (2014). Briefly, 150 µL VCl_3 reagent (2% w/v VCl_3 in 6M HCl) and 1 ml sample of KCl extract were combined in 1.5-ml reaction tubes, mixed, and incubated at 60 ºC for exactly 100 minutes and immediately chilled on ice. Combined NO_2^- and NO_3^- was subsequently determined colorimetrically using the modified sulfanilamide-NED reagent as described [81].

Net nitrification and N_2O production measurements

Nitrification rates and N_2O production rates were determined in soil microcosms. Microcosms were constructed from 125 ml food-grade glass jars with gas-tight lids (Specialty Bottle, Seattle, WA). Lids were modified with ¼ inch× ¼ inch brass bulkhead

reducers (Ham-Let, Missouri City, TX) and sealed with 5 mm Thermolite plus Septa (Restek Corporation, Bellefonte, PA). Four days after field sampling, triplicate microcosms were set up for each sub-plot sample by adding 10 g of fresh field-moist soil to each microcosm. Microcosms were pre-incubated at near daily average surface soil temperature of 28 °C in darkness for 48 hours and time series were started by removing 0.5 g soil sample (T0) and equilibration of the headspace to ambient air before sealing each microcosm. Headspace samples (2 ml) for gas analysis and soil samples (0.5 g) for nutrient analyses were collected after 24, 48, and 144 h. Nutrients were analyzed using colorimetric assays as described above and net nitrification rates were calculated from linear regression of NO_2^- + NO_3^- over time.

Greenhouse gas analyses

Gas samples collected from microcosms were analyzed directly by manual injection using a greenhouse gas chromatograph (SRI Instruments, Las Vegas, NV) equipped with 1-ml sample loop, and FID-methanizer and electron capture detectors, respectively. Gases were separated on two sequential 2m HayeSep D columns with vent valve configuration at 80 °C. The instrument was calibrated between 0 and 4,300 ppb N_2O, 0-18 ppm CH_4, and 0- 4,000 ppm CO_2 using NIST-traceable certified mixed standards of CO_2, CH_4, and N_2O in N_2 (Praxair, Durham, NC) and reproducibility was better than +/-3%. Carbon mineralization and N_2O production rates were calculated by linear regression of cumulative gas accumulation over the 144 h incubations.

Nitrification potentials

On day four after returning from the field, triplicate 1.0-g samples of field-moist soil were added to 20 ml SFCM media in 50-ml Falcon tubes. The SFCM media contained 1 g l^{-1} NaCl, 0.4 g l^{-1} $MgCl_2$*6 H_2O, 0.1 g l^{-1} $CaCl_2$ *$2H_2O$ and 0.5 g l^{-1} KCl in double deionized water (EMD Millipore Synergy UV) [82]. After autoclaving the media was cooled to room temperature and supplemented with the following sterile stock solutions (per liter): 2 ml $NaHCO_3$ (84 g l^{-1}), 5 ml KH_2PO_4 (0.4 g l^{-1}), 1ml FeNaEDTA (2.75 g l^{-1}), 1 ml modified non-chelated trace element solution [70]. The medium was buffered at pH 7.2 using ~2 mM sodium bicarbonate and 1 mM NH_4Cl was added as source of nitrogen. Samples were vortexed briefly and incubated alongside net nitrification samples at 28 ºC. Samples (1.0 ml) for determination of NO_2^- + NO_3^- were collected at 0 h, 24 h, 48 h, and 168 h of incubation. Nutrient concentrations were determined using colorimetric assays as described above and potential rates were determined from linear regression of NO_2^- + NO_3^- accumulation over the first 48 h of incubation.

Clone library analysis of archaeal, bacterial and comammox *amoA*

Amplicon clone libraries were generated for each plot and ammonia oxidizer group. Gene fragments of target *amoA* were PCR-amplified using previously described primers for archaeal *amoA* [41], bacterial *amoA* [42] and comammox *amoA* clade A and clade B [20]. PCR reactions (25 µL) contained 12.5 µl of GoTaq PCR Mastermix (Promega, Fitchburg, WI), 1 µl each of forward and reverse primer (0.5 µM final concentration for archaeal and bacterial *amoA*, 0.25 µM final each for comammox *amoA*) and 2 µl of DNA

template (~5ng/μl). Annealing temperatures were optimized empirically for each primer set to the lowest temperature yielding specific amplicons of expected size. Final PCR cycling conditions were as follows: Initial denaturation at 94 °C for 5 min, followed by 30 cycles of 30 sec denaturation at 94, 30 seconds at 52 °C annealing (53 °C for comammox *amoA*), 45 seconds at 72 °C primer extension and 10 min final extension. Clone library analysis (see below) revealed no clones of comammox *amoA* in amplicons from Plot 1. PCR reactions and cloning were therefore repeated using amplicons generated by the qPCR reagents and procedure (see below) with 35 amplification cycles. All PCR products were electrophoretically separated on 1.5% agarose gels, cut out and purified by QIAquick Gel Extraction Kit kits (Qiagen, MD, USA). Purified DNA was quantified by NanoDrop and at least three PCR reactions for each sample were pooled in equimolar ratios, cloned into TOPO TA PCR2.1 vectors and transformed into OneShot TOP10 chemically competent *E. coli* following kit instructions (Thermo Fisher Scientific, MA, USA), except ligation reactions were carried out overnight for best cloning efficiency. Following blue-white screening and PCR-checking, 95 clones per library were grown up overnight in Lysogeny broth (LB) plus 40 μg/ml kanamycin. Plasmids were extracted using the QIAprep Spin Miniprep Kit (Qiagen, MD, USA) and inserts were sequenced at Eurofins Genomics (Huntsville, AL). Sequences were trimmed using sangerseqR package [43] and then imported into ARB program [44], aligned and manually checked. An initial ARB-formatted alignment of comammox *amoA* gene sequences was provided by Pjevac et al. [20] and amended with new sequences obtained from NCBI genbank. Archaeal and bacterial *amoA* gene ARB databases were built from sequences obtained from NCBI genbank. Neighbor-joining and Maximum-likelihood phylogenetic trees were

calculated in ARB using the DNA distance method with Jukes-Cantor correction and RAxML algorithm with GTRGAMMA-25 rate distribution model and rapid hill climbing algorithm, respectively, based on 595, 453, and 383 nucleotide positions for archaeal, bacterial, and comammox *amoA*, respectively. QIIME 1.9.1 [45] was employed to group sequences into operational taxonomic units (OTU) with a range of sequence similarity from 85%-99% relative to known AOA and AOB and comammox strains, and 96%, 97% and 94% sequence similarity were chosen for archaeal, bacterial, and comammox *amoA*, respectively, and applied to phylogenetic trees shown.

16S rRNA gene amplicon library preparation and sequencing

PCR amplifications, library preparations and DNA sequencing were conducted at the Environmental Sample Preparation and Sequencing Facility (ESPSF) at Argonne National Laboratory. PCR products for 16S rRNA gene amplicon libraries were generated following Earth Microbiome Project protocols [46]. The V4 region of the 16S rRNA gene was amplified using primers 515F [47] and 806R [48]. Forward primer included sequencing adapter sequences and reverse primer contained the twelve base barcode sequence. PCR reactions contained 9.5 µL certified DNA-Free PCR Water (MoBio, USA), 12.5 µL 2x AccuStart II PCR ToughMix (QuantaBio, Beverly, MA), 1 µL 200 pM forward primer, 200 pM Golay barcode-tagged reverse primer, and 1 µL template DNA. PCR protocol included denaturation at 94 °C for 3 minutes followed by 35 cycles at 94 °C for 45 s, 50 °C for 60 s, and 72 °C for 90 s; with a final extension of 10 min at 72 °C. PCR products were quantified using PicoGreen (Invitrogen, Carlsbad, CA) in a 96-well microplate reader (Infinite® 200 PRO, Tecan, Grödig, Austria). PCR

products were pooled in equimolar amounts and purified using AMPure XP Beads (Beckman Coulter, Brea, CA), quantified by Qubit DNA quantification kit (Invitrogen, Carlsbad, CA), diluted to 2 nM, denatured, and then diluted to 6.75 pM final concentration with 10% PhiX spike. Sequencing was performed on a Illumina MiSeq instrument using 150 cycle MiSeq Reagent kit v3 (Illumina, San Diego, Ca).

Amplicon sequences were demultiplexed on the instrument. Subsequent initial sequence analyses including barcode removal, quality filtering, trimming, read merging and chimera screening were conducted using DADA2 [49] with default settings implemented in the QIIME 2 package [50]. The representative sequences and amplicon sequence variants (ASV) table generated in the DADA2 denoising step served as inputs for subsequent taxonomic assignments via qiime feature-classifier using sklearn algorithm against the Silva database version 132 [51]. ASV sequences were imported into ARB, manually aligned to 16S rRNA gene reference sequences (> 1,400 nt). Backbone phylogenetic trees were calculated for *Thaumarchaeota* and *Nitrospira* sp., respectively, using maximum-likelihood method aaAxML rate distribution model and maximum base frequency filters for *Archaea* and *Nitrospirae*, respectively. Short sequences were inserted into the trees using the “quick add sequences by parsimony” option in ARB using respective filters and position limits according to *E. coli* position 534 and 786.

Shannon Index, Observed_OTUs, Faith’s phylogenetic diversity and Bray-Curtis dissimilarity matrices were calculated using “qiime diversity core-metrics-phylogenetic” command in Qiime 2 after rarefaction to 25,900 sequences (whole microbial community 16S rRNA) and 780 sequences (thaumarchaeal 16S rRNA). The Bray-Curtis distance

matrix of thaumarchaeal *amoA* was calculated using the vegdist function of the vegan package (https://CRAN.R-project.org/package=vegan [52]) implemented in R, after rarefying to 53 sequences. Subsequent principal coordinate analyses (PCoA), canonical correspondence analyses (CCA), and distance-based redundancy analysis (dbRDA) were run by using the pcoa, cca, and capscale functions in vegan, respectively. Significance of differences between plots were tested by the adonis function with 999 permutations in vegan. Pearson correlations and ANOVA comparison of means were conducted in base R version 3.6.3 [53].

Quantification of *amoA* gene copy numbers by quantitative PCR

Ammonia monooxygenase subunit A *(amoA)* genes of archaeal, betaproteobacterial, and comammox Nitrospira were quantified by real-time PCR (qPCR) using primers for archaeal *amoA* [41], bacterial *amoA* [42] and comammox *amoA* clade A and clade B [20] using the QuantiTect SYBR® Green qPCR kit (Qiagen, Germantown, MD) in a Bio-Rad IQ5 real-time PCR system (Bio-Rad, Hercules, CA). The 20 µL PCR reactions contained 10 µl of QuantiTect SYBR® Green Master Mix, 1 µl each of forward and reverse primer (10 µM for archaeal and bacterial *amoA*, 5 µM each for combined comammox *amoA*), and 2 µl of DNA template (5 ng/µl). Genomic DNA isolated from *Nitrososphaera viennensis* strain *EN 76* [10] and *Nitrosospira briensis* Nsp10 was used as qPCR standards for archaeal and betaproteobacterial *amoA,* respectively*.* A comammox *amoA* standard was made from a comammox *amoA* clone plasmid carrying the most abundant sequence type found in our samples (OTU11). The plasmid was linearized by overnight restriction digest with NotI (New England Biolabs, MA, USA), purified by DNA

precipitation, and complete digestion confirmed by gel electrophoresis. All qPCR standards were stored in aliquots at -80 °C until use.

Annealing temperatures for all qPCR assays were optimized in temperature gradients using soil DNA samples and serial dilutions of standards (10-10^6 *amoA* copies/ul). The optimized PCR cycling conditions were as follows: Initial denaturation at 94 °C for 5 min, 40 cycles of 45 sec denaturation at 94 °C, 30 seconds annealing at 54 °C, 58 °C, and 53 °C for archaeal *amoA*, bacterial *amoA*, and comammox *amoA*, respectively, and 45 seconds at 72 °C extension, followed by a detection step 15 seconds at 80 °C. Melting curve analysis was run in 0.5 °C increments every 10 s between 60 °C and 95 °C. PCR products were checked by gel electrophoresis. Gene copy numbers were calculated and transformed to copies/g dry weight soil. Under the given qPCR conditions, the PCR amplification efficiencies were 86% for archaeal *amoA*, 90% for bacterial *amoA* and 96% for comammox *amoA.*

Supplementary Results

Soil physicochemical characterization

In order to examine the response of soil microbial communities to agricultural crops and management we examined four soil plots with different recent crop cover and an unmanaged control plot in close proximity to each other in the Everglades Agricultural Area (Supplementary Figure 1).

Soil moisture ranged from of 0.39± 0.01 to 0.47± 0.01 % (*w/w*) with significantly higher values in plots 3 and 5 ($P < 0.05$). As expected, following long-term agricultural management pH was significantly higher (pH ≥ 7) in agricultural plots than in the native plot 1 (pH < 6.0; $P < 0.05$) and slightly increasing from plot 2 to 5.

High concentrations of NO_3^--N (43.8±9.35 to 116.10 ± 70.31 µg/g) were observed in plots 1 to 4, with highest NO_3^--N concentrations in plots with significant fallow periods (Figure 1A). In contrast, plot 5 from which sugar cane had been harvested only about one week before sampling, NO_3^--N of 2.99± 0.49 µg/g, was approximately 15-time lower than in unmanaged plot 1 and 23- to 38-fold lower than in the plots with significant fallow periods ($P < 0.05$; Figure 1).

Concentrations of NH_4^+-N (5.66 ± 0.43 to 8.56 ± 0.68 µg/g) were slightly higher in plots with recent plant cover. However, there were no statistically significant differences between plots. Total and available P followed similar trends. TP ranged from 1.20±0.04 g/kg in plot 1 up to 1.93±0.15 g/kg in plot 2, with plot 3 showing only slightly higher values than plot 1, and plot 4 and 5 showing intermediate concentrations). Available P ranged from 22.00±3.61 mg/kg in plot 1 to 117.67±8.14 mg/kg in plot 2. Dissolved organic carbon (DOC) concentrations were highest in plot 1 (5871.01 ± 200.50 mg/kg)

and plot 5 (4784.15 ± 99.67 mg/kg) and slightly lower in plot 2-4 (2911.97 ± 133.57 mg/kg to 4040.19 ± 193.00 mg/kg) (Supplementary Figure 2). Total organic carbon (TOC) was highest in the unmanaged plot (82.72 ± 1.09 %) and between 5.31 and 12.56% lower in the managed plots. Altogether, the results suggested that the type of plant cover and length of fallow periods significantly influenced soil physicochemical properties.

Overall microbial community composition

Among the total 990,311 high-quality paired-end sequences (66,011 ± 23,456 sequences per sample from the 15 EAA soil samples), a total of 12,179 ASV's were identified and phylogenetically affiliated with 2,420 distinct species-level taxa within 55 bacterial and archaeal phyla. *Archaea* ranged between 2.48 ± 0.55% of all sequences in plot 1 and 6.65 ± 0.85% in plot 2, with intermediate numbers around 4.0% and no significant differences between plots 3 – 5. Non-thaumarchaeotal sequences constituted a minor fraction (range 0.06 ± 0.81% to 0.37 ± 0.86%). Conversely, bacterial sequences ranged between 93.3±1.0% in plot 2 and 97.5±0.7% in plot 1. As expected, only a marginal fraction of sequences (0.01%) was affiliated with Eukaryotes in all samples. Overall, bacterial sequences associated with 11 major phyla commonly found in soil: *Proteobacteria* (averaging 32.5% of all sequences in all plots), *Acidobacteria* (17.4%), *Actinobacteria* (12.5%), *Chloroflexi* (7.4%), *Planctomycetes* (5.0%), *Bacterioidetes* (4.4%), *Gemmatimonadetes* (3.4%), *Verrucomicrobia* (2.9%), *Firmicutes* (2.9%), *Rokubacteria* (1.9%), *Nitrospirae* (1.3%). Sequences associated with all other less frequent phyla totaled 4.2%.

Supplementary References

75. Lodge TE (2016). *The Everglades Handbook: Understanding the ecosystem*. CRC Press: Boca Raton.

76. Entry JA, Gottlieb AD, Jayachandran K, Ogram A (eds) (2015). *Microbiology of the Everglades ecosystem*. CRC Press: Boca Raton.

77. Snyder G (2005). Everglades agricultural area soil subsidence and land use projections. *Soil Crop Science Society Florida Proceedings.* **64:** 44-51.

78. Kadlec RH (2009). Everglades and agriculture in South Florida. In: Maltby E, Barker T (eds). *The Wetlands Handbook*. Wiley-Blackwell.

79. Bower CE, Holm-Hansen T (1980). A salicylate–hypochlorite method for determining ammonia in seawater. *Can J Fish Aquat Sci.* **37:** 794-798.

80. Grasshoff K, Kremling K, Erhard M (1999). *Methods of seawater analysis*, 3 edn. Wiley-VCH.

81. García-Robledo E, Corzo A, Papaspyrou S (2014). A fast and direct spectrophotometric method for the sequential determination of nitrate and nitrite at low concentrations in small volumes. *Mar Chem.* **162:** 30-36.

82. de la Torre JR, Walker CB, Ingalls AE, Könneke M, Stahl DA (2008). Cultivation of a thermophilic ammonia oxidizing archaeon synthesizing crenarchaeol. *Environ Microbiol.* **10:** 810-818.
