# Supplementary material for: Ammonia-oxidizing archaea are integral to nitrogen cycling in a highly fertile agricultural soil
Source: ISME Commun. 2021 Jun 1;1:19. doi: 10.1038/s43705-021-00020-4 (PMC9723749; doi:10.1038/s43705-021-00020-4)
Supplement: Supplementary file 2 — Supplemental Figures 1–7 [file 43705_2021_20_MOESM2_ESM.pdf]

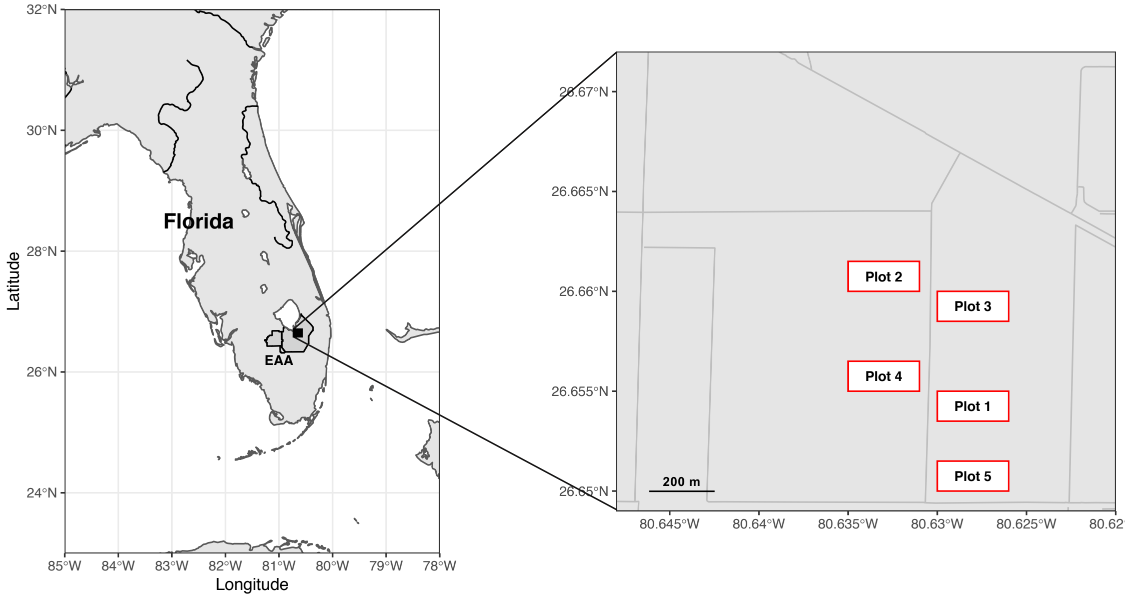

Supplementary Figure 1. Sampling location in the Everglades Agricultural Area (EAA) south of Lake Okeechobee. The area is characterized by rich and fertile histosol soils originally obtained after draining freshwater sawgrass marshes of the Everglades wetlands early last century.

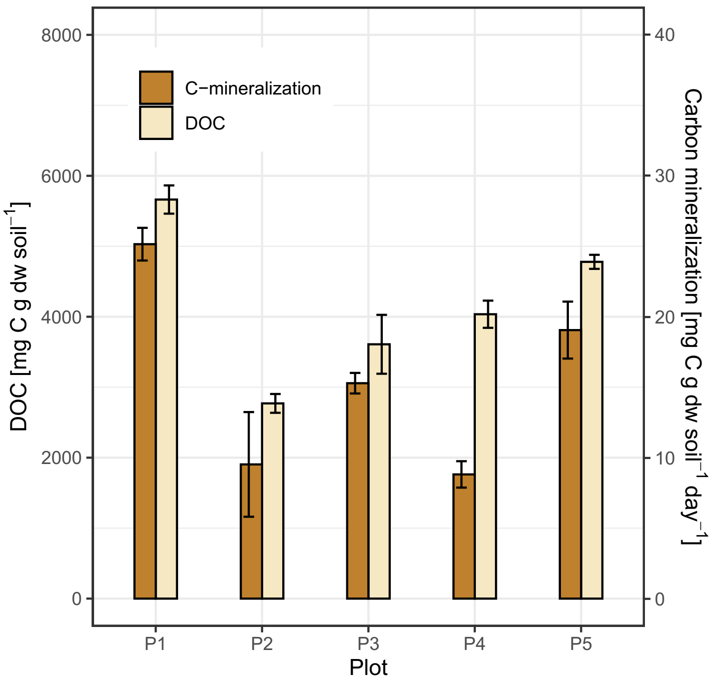

Supplementary Figure 2. DOC concentrations and carbon mineralization rates of EAA soils. Given are means and standard deviations of DOC concentrations (n =3), and carbon mineralization (n= 9 replicates).

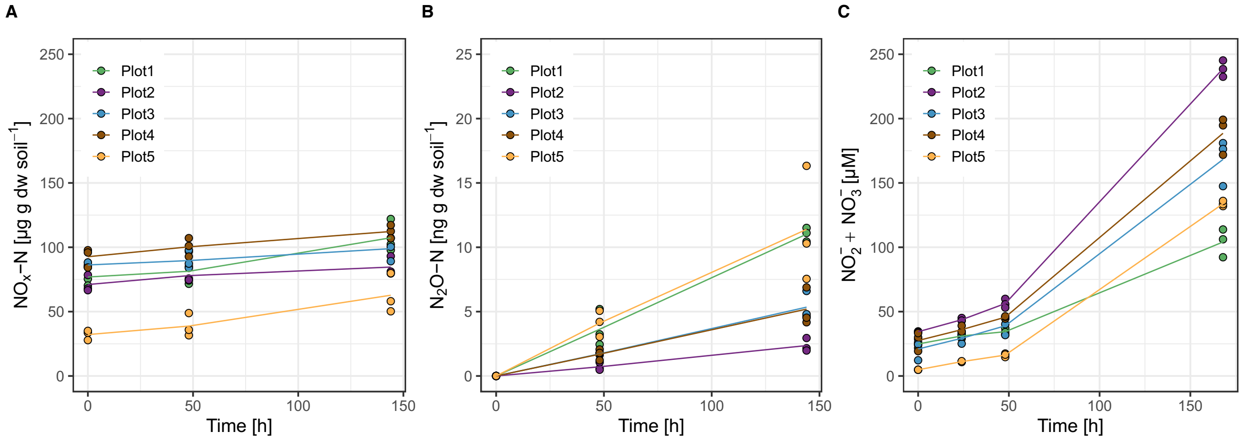

Supplementary Figure 3. Time course of NO_x-N (= NO_2^- + NO_3^-) accumulation (A) and corresponding N_2O accumulation (B) in net nitrification incubations, and NO_x-N accumulation in nitrification potential incubations (C). Each point represents the mean of triplicate incubations. Error bars of triplicate incubations were small and neglected for clarity. Net nitrification rates and N_2O production rates were calculated from linear regression of NO_2^- + NO_3^- and N_2O accumulation during 144 h incubations. Nitrification potentials were cacluated from linear regression of the first 48h of incubations.

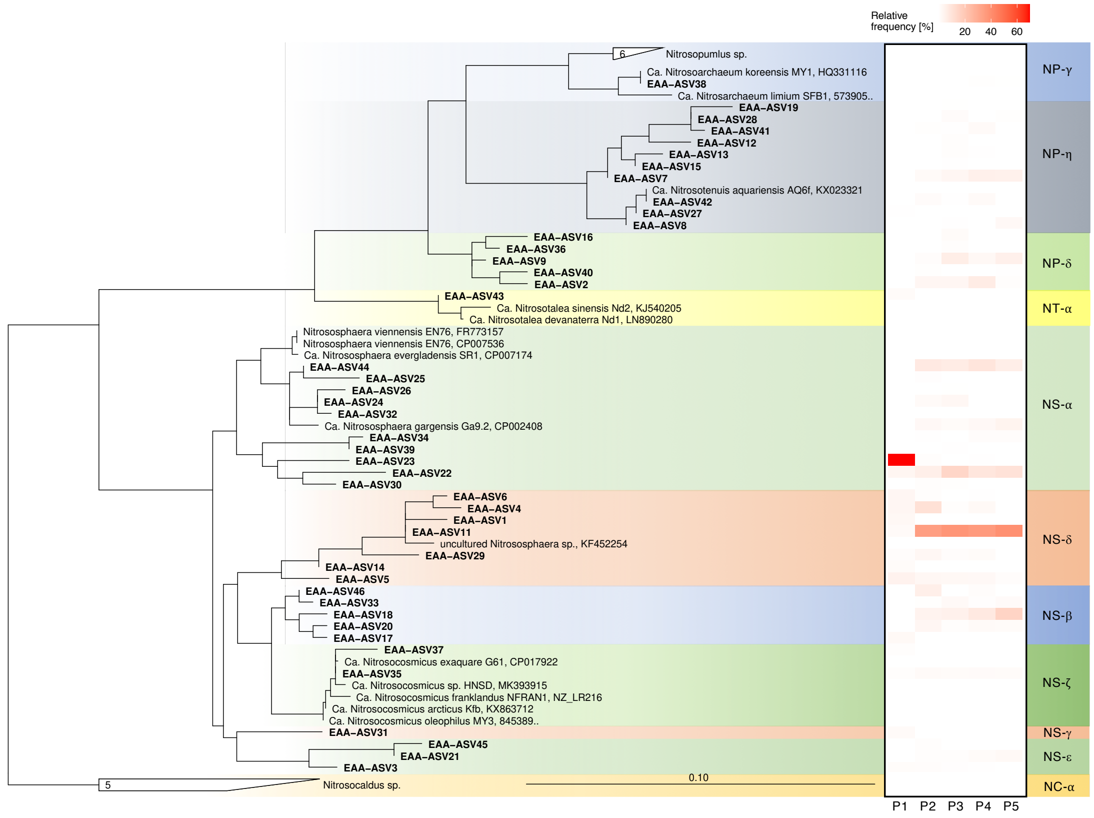

Supplementary Figure 4. Maximum-likelihood phylogenetic tree displaying thaumarchaeal 16S rRNA gene amplicon sequence affiliations and relative abundance heatmap of each ASV across EAA plots 1 to 5. The backbone phylogenetic tree was calculated using near full-length sequences (>1,400 nt) and ASV sequences were inserted using “quick add sequences using parsimony” option in ARB using positional variability filter limited to *E. coli* positions 534 and 786.

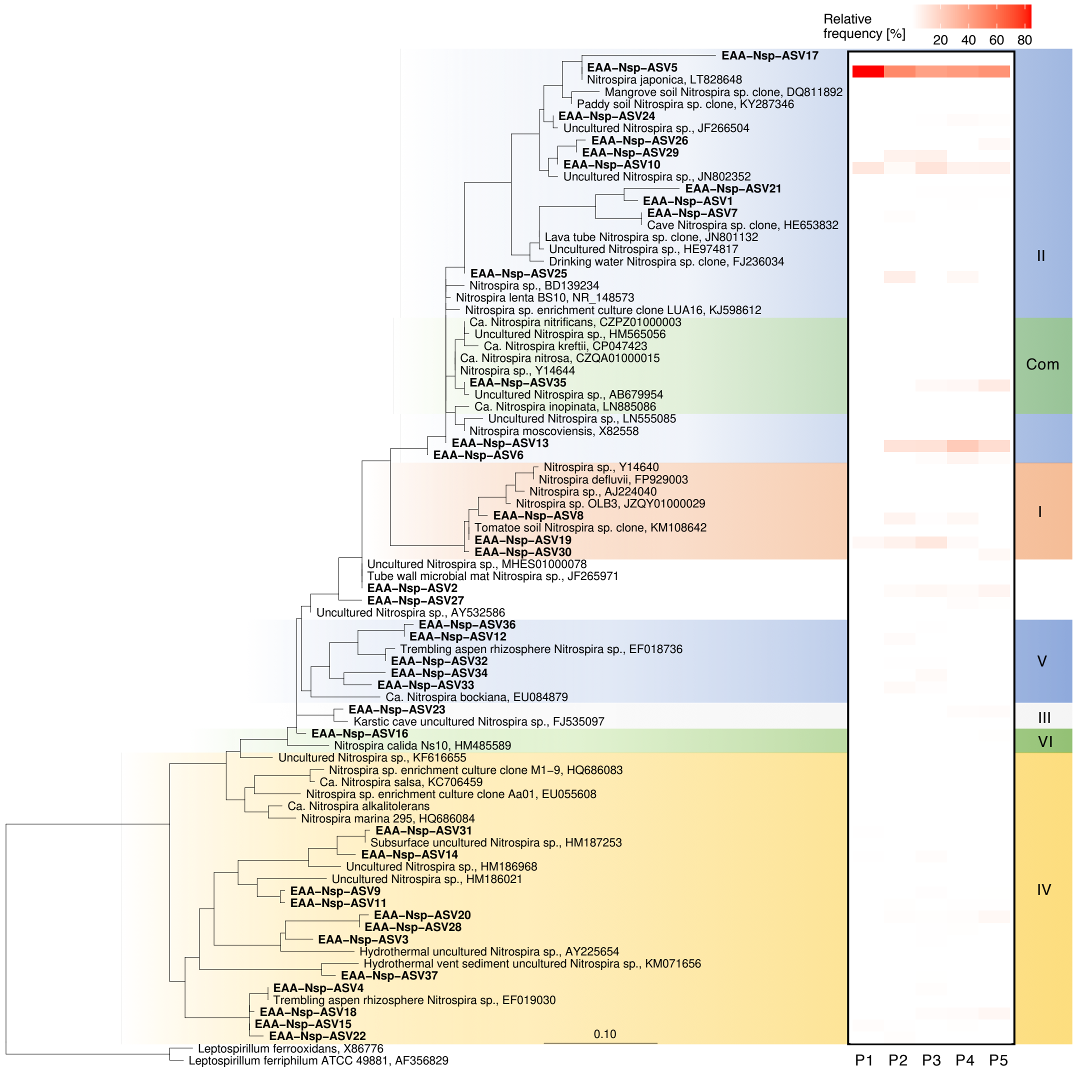

Supplementary Figure 5. Maximum-likelihood phylogenetic tree displaying *Nitrospira* sp.-affiliated 16S rRNA gene ASV sequences obtained from EAA plot 1 – 5 and relative abundance heatmap of each ASV. The backbone phylogenetic tree was calculated using near full-length sequences (>1,400 nt) and ASV sequences were inserted using "quick add sequences using parsimony" option in ARB using positional variability filter limited to *E. coli* positions 534 and 786.

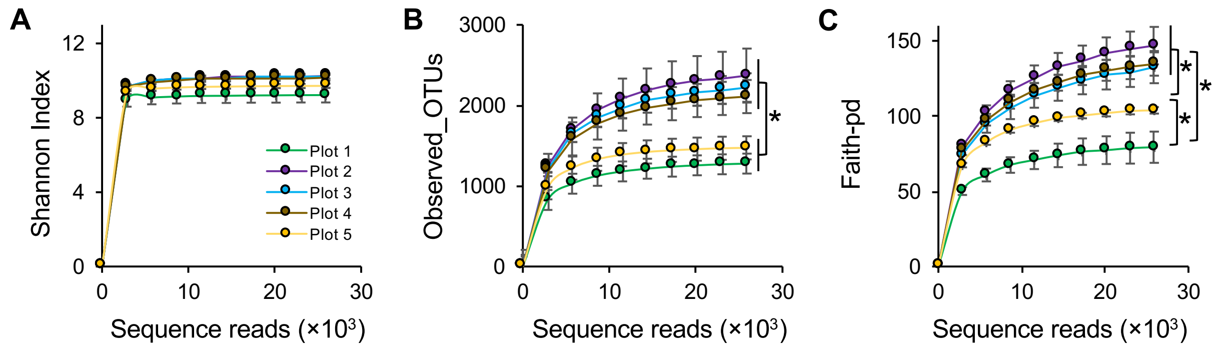

Supplementary Figure 6. Alpha diversity metrics of EAA microbial communities based on 16S rRNA gene sequences. (A) Shannon Index, (B) Observed OTUs, and (C) Faith's phylogenetic diversity. Error bars denote standard deviations between sub-plots and * indicate significant differences between plots (ANOVA with Tukey HSD test at α = 0.05 level).

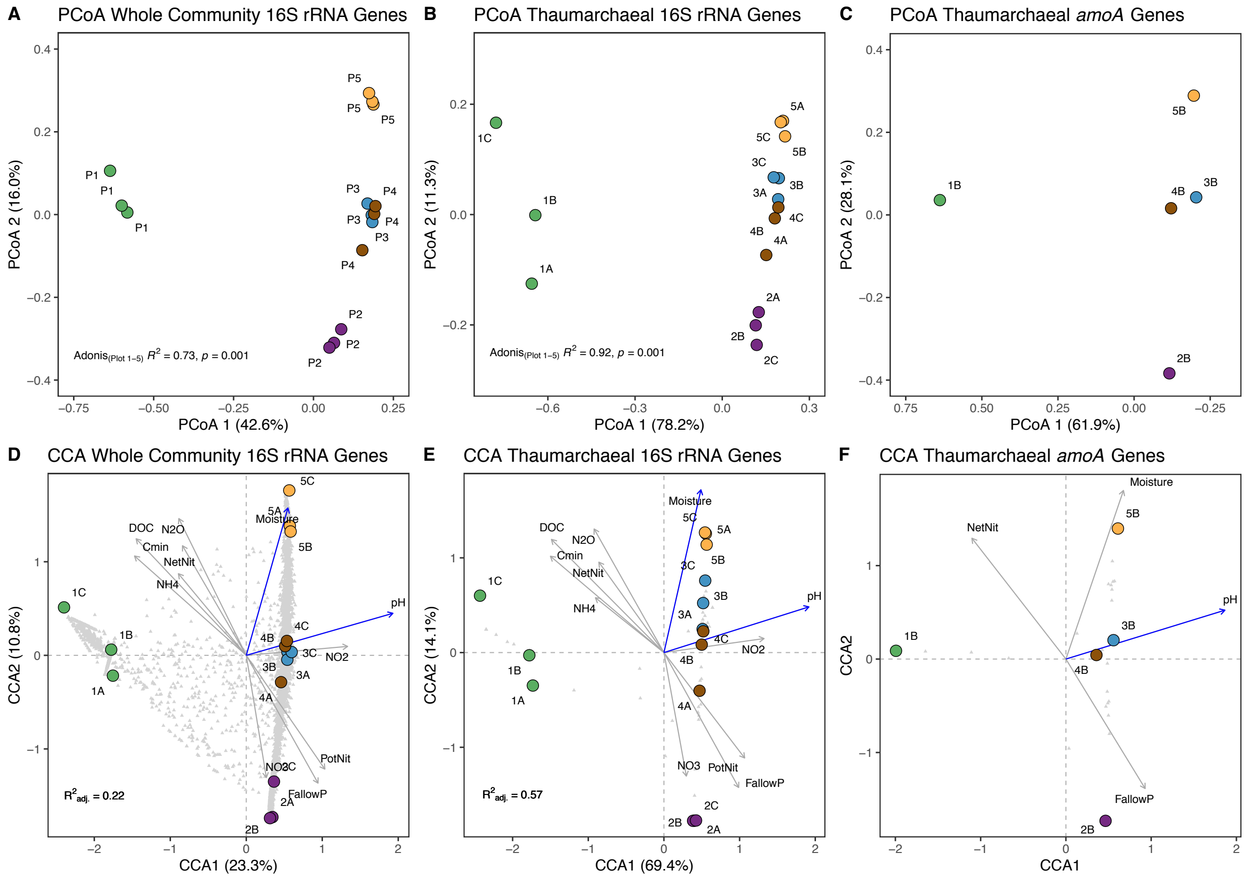

Supplementary Figure 7. Principal coordinate analysis (PCoA) (A-C) and canonical correspondence analysis (CCA) (D-F) of EAA microbial communities based on 16S rRNA and *amoA* gene sequences. Shown are diversity patterns of total microbial communities (A, D), thaumarchaeal 16S rRNA genes only (B, E), and archaeal *amoA* gene sequences. Arrows denote explanatory factors tested. Significant factors are highlighted in blue ($P < 0.01$). Inclusion of more explanatory variables did not improve the models (pairwise ANOVA between two and three factor models).
